# Some Properties of Solutions of a Functional-Differential Equation of Second Order with Delay

**DOI:** 10.1155/2014/878395

**Published:** 2014-02-10

**Authors:** Veronica Ana Ilea, Diana Otrocol

**Affiliations:** ^1^Babeş-Bolyai University, Kogălniceanu no. 1, RO-400084 Cluj-Napoca, Romania; ^2^“T. Popoviciu” Institute of Numerical Analysis, Romanian Academy, P.O. Box. 68-1, 400110 Cluj-Napoca, Romania

## Abstract

Existence, uniqueness, data dependence (monotony, continuity, and differentiability with respect to parameter), and Ulam-Hyers stability results for the solutions of a system of functional-differential equations with delays are proved. The techniques used are Perov's fixed point theorem and weakly Picard operator theory.

## 1. Introduction

Functional-differential equations with delay arise when modeling biological, physical, engineering, and other processes whose rate of change of state at any moment of time *t* is determined not only by the present state but also by past state.

The description of certain phenomena in physics has to take into account that the rate of propagation is finite. For example, oscillation in a vacuum tube can be described by the following equation in dimensionless variables [1, 2]:

(1) $$\begin{array}{c} x^{\prime \prime }\left(t\right)+2rx^{\prime }\left(t\right)+\omega^{2}x\left(t\right)+2qx^{\prime }\left(t-1\right)=\epsilon x^{\prime 3}\left(t-1\right). \end{array}$$

In this equation, time delay is due to the fact that the time necessary for electrons to pass from the cathode to the anode in the tube is finite. The same equation has been used in the theory of stabilization of ships [2]. The dynamics of an autogenerator with delay and second-order filter was described in [3] by the equation

(2) $$\begin{array}{c} x^{\prime \prime }\left(t\right)+2\delta x^{\prime }\left(t\right)+x\left(t\right)=f\left(x\left(t-h\right)\right). \end{array}$$

The model of ship course stabilization under conditions of uncertainty may be described by the following equation [4]:

(3) Ix′′(t)+Hx′(t)=−KΨ(t)+b0ξ0′(t),x(t0)=x0, x′(t0)=0,

with *x*(*t*) being the angle of the deviation from course, Ψ(*t*) the turning angle of the rudder, and *ξ*_0_(*t*) the stochastic disturbance. In the process of mathematical modeling, often small delays are neglected; that is why sometimes false conclusions appear. As an example we can give the following equation [1]:

(4) x′′(t)+x′(t)+x(t) =a[x′′(t−h)+x′(t−h)+x(t−h)],

which is asymptotically stable for *h* = 0 but unstable for arbitrary *h* > 0. Here *a* > 1. If *h* = 0 the above system is asymptotically stable. The characteristic equation is Δ(*z*) = (*z* ^2^ + *z* + 1)(1 − *ae* ^−*hz*^) and has the following zeros with positive real part if *h* > 0 : *z*_*k*_ = 1/*h*(In⁡*a* + 2*kπi*), *i* ^2^ = −1, *k* = 0, ±1,…. So the trivial solution is unstable for any *h* > 0.

In this paper, we continue the research in this field and develop the study of the following general functional differential equation with delay:

(5) x′′(t)=f(t,x(t),x′(t),x(t−h),x′(t−h)),t∈[a,b],x(t)=φ(t), t∈[a−h,a].

Existence, uniqueness, data dependence (monotony, continuity, and differentiability with respect to parameter), and Ulam-Hyers stability results of solution for the Cauchy problem are obtained. Our results are essentially based on Perov's fixed point theorem and weakly Picard operator technique, which will be presented in Section 2. More results about functional and integral differential equations using these techniques can be found in [5–8]. The problem (5) is equivalent to the following system:

(6) x′(t)=z(t), t∈[a,b],z′(t)=f(t,x(t),z(t),x(t−h),z(t−h)),t∈[a,b],

with the initial conditions

(7) $$\begin{array}{c} x\left(t\right)=\varphi \left(t\right),\;t\in \left[a-h,a\right],\\ z\left(t\right)=\varphi^{\prime }\left(t\right),\;t\in \left[a-h,a\right]. \end{array}$$

By a solution of the system (6) we understand a function $\left(\begin{array}{c} x\\ z \end{array})\in C([a-h,b],{\mathbb{R}}^{2})\cap C^{1}([a,b],{\mathbb{R}}^{2}\right)$ that verifies the system.

We suppose that

(C_1_) *a* < *b*, *h* > 0;
(C_2_) *f* ∈ *C*([*a*, *b*] × ℝ^4^, ℝ), *φ* ∈ *C* ^1^([*a* − *h*, *a*], ℝ);
(C_3_) there exists *L*_1_, *L*_2_ > 0 such that ∀*t* ∈ [*a*, *b*], $u_{i},v_{i},{\tilde{u}}_{i},{\tilde{v}}_{i}\in \mathbb{R}$ , *i* = 1,2, we have
(8) |f(t,u1,v1,u2,v2)−f(t,u~1,v~1,u~2,v~2)| ≤L1 max(|u1−u~1|,|u2−u~2|)  +L2 max(|v1−v~1|,|v2−v~2|).

If $\left(\begin{array}{c} x\\ z \end{array})\in C([a-h,b],{\mathbb{R}}^{2})\cap C^{1}([a,b],{\mathbb{R}}^{2}\right)$ is a solution of the problem (6)-(7), then $\left(\begin{array}{c} x\\ z \end{array}\right)$ is a solution of the following integral system:

(9) (xz)(t)={(φφ′)(t),for  t∈[a−h,a],(φ(a)+∫atz(s)dsφ′(a)+∫atf(s,x(s),z(s),x(s−h),z(s−h))ds)for  t∈[a,b].

If $\left(\begin{array}{c} x\\ z \end{array})\in C([a-h,b],{\mathbb{R}}^{2}\right)$ is a solution of (9), then $\left(\begin{array}{c} x\\ z \end{array})\in C([a-h,b],{\mathbb{R}}^{2})\cap C^{1}([a,b],{\mathbb{R}}^{2}\right)$ and $\left(\begin{array}{c} x\\ z \end{array}\right)$ is a solution of (6)-(7).

Moreover, the system (6) is equivalent to the functional integral system

(10) (xz)(t)={(xz)(t),for  t∈[a−h,a],(x(a)+∫atz(s)dsz(a)+∫atf(s,x(s),z(s),x(s−h),z(s−h))ds)for  t∈[a,b].

We consider the operators *B*, *E* : *C*([*a* − *h*, *b*], ℝ^2^) → *C*([*a* − *h*, *b*], ℝ^2^), $B=(\begin{array}{c} B_{1}(\begin{array}{c} x\\ z \end{array})\\ B_{2}(\begin{array}{c} x\\ z \end{array}) \end{array})$ , $\;E=(\begin{array}{c} E_{1}(\begin{array}{c} x\\ z \end{array})\\ E_{2}(\begin{array}{c} x\\ z \end{array}) \end{array})$ defined by $B(\begin{array}{c} x\\ z \end{array})(t)≔$ the right hand side of (9), for *t* ∈ [*a* − *h*, *b*] and $E(\begin{array}{c} x\\ z \end{array})(t)≔$ the right hand side of (10), for *t* ∈ [*a* − *h*, *b*].

## 2. Preliminaries

In this section, we introduce notations, definitions, and preliminary results which are used throughout this paper; see [9–17]. Let (*X*, *d*) be a metric space and *A* : *X* → *X* an operator. We will use the following notations:

 
*F*_*A*_≔{*x* ∈ *X* | *A*(*x*) = *x*}—the fixed points set of *A*;
 
*I*(*A*)≔{*Y* ⊂ *X* | *A*(*Y*) ⊂ *Y*, *Y* ≠ *∅*}—the family of the nonempty invariant subset of *A*;
 
*A* ^*n*+1^≔*A*∘*A* ^*n*^, *A* ^0^ = 1_*X*_, *A* ^1^ = *A*, *n* ∈ *ℕ*.

**Definition 1**

Let (*X*, *d*) be a metric space. An operator *A* : *X* → *X* is a Picard operator (PO) if there exists x* ∈ *X* such that

- *F*_*A*_ = {x*};
- the sequence (*A* ^*n*^(*x*_0_))_*n*∈*ℕ*_ converges to x* for all *x*_0_ ∈ *X*.

**Definition 2**

Let (*X*, *d*) be a metric space. An operator *A* : *X* → *X* is a weakly Picard operator (WPO) if the sequence (*A* ^*n*^(*x*))_*n*∈*ℕ*_ converges for all *x* ∈ *X* and its limit (which may depend on *x*) is a fixed point of *A*.

**Definition 3**

If *A* is a weakly Picard operator, then we consider the operator *A* ^*∞*^ defined by

(11) $$\begin{array}{c} A^{\infty }:X⟶X,\;A^{\infty }\left(x\right)≔\underset{n\to \infty }{\lim}A^{n}\left(x\right). \end{array}$$

**Remark 4**

It is clear that *A* ^*∞*^(*X*) = *F*_*A*_.

**Definition 5**

Let *A* be a weakly Picard operator and *c* > 0. The operator *A* is *c*-weakly Picard operator if

(12) $$\begin{array}{c} d\left(x,A^{\infty }\left(x\right)\right)\leq cd\left(x,A\left(x\right)\right),\;\forall x\in X. \end{array}$$

The following concept is important for our further considerations.

**Definition 6**

Let (*X*, *d*) be a metric space and *f* : *X* → *X* an operator. The fixed point equation

(13) $$\begin{array}{c} x=f\left(x\right) \end{array}$$

is Ulam-Hyers stable if there exists a real number *c*_*f*_ > 0 such that for each *ɛ* > 0 and each solution y* of the inequation

(14) $$\begin{array}{c} d\left(y,f\left(y\right)\right)\leq ɛ, \end{array}$$

there exists a solution x* of (13) such that

(15) $$\begin{array}{c} d\left(y^{*},x^{*}\right)\leq c_{f}ɛ. \end{array}$$

Now we have the following.

**Theorem 7 (see [17])**

If *f* : *X* → *X* is *c*-WPO, then the equation

(16) $$\begin{array}{c} x=f\left(x\right) \end{array}$$

is Ulam-Hyers stable.

Another result from the WPO theory is the following (see, e.g., [11]).

**Theorem 8 (fibre contraction principle)**

Let (*X*, *d*) and (*Y*, *ρ*) be two metric spaces and *A* : *X* × *Y* → *X* × *Y*, *A* = (*B*, *C*), (*B* : *X* → *X*, *C* : *X* × *Y* → *Y*) a triangular operator. One supposes that

- (*Y*, *ρ*) is a complete metric space;
- the operator *B* is Picard operator;
- there exists *l* ∈ [0,1) such that *C*(*x*, ·) : *Y* → *Y* is a *l*-contraction, for all *x* ∈ *X*;
- if (x*, y*) ∈ *F*_*A*_, then *C*(·, y*) is continuous in x*.

Then the operator *A* is Picard operator.

Throughout this paper we denote by *M*_*mm*_(ℝ_+_) the set of all *m* × *m* matrices with positive elements and by *I* the identity *m* × *m* matrix. A square matrix *Q* with nonnegative elements is said to be convergent to zero if *Q* ^*k*^ → 0 as *k* → *∞*. It is known that the property of being convergent to zero is equivalent to each of the following three conditions (see [9, 10]):

- *I* − *Q* is nonsingular and (*I* − *Q*)^−1^ = *I* + *Q* + *Q* ^2^ + ⋯ (where *I* stands for the unit matrix of the same order as *Q*);
- the eigenvalues of *Q* are located inside the open unit disc of the complex plane;
- *I* − *Q* is nonsingular and (*I* − *Q*)^−1^ has nonnegative elements.

We finish this section by recalling the following fundamental result (see [9, 18]).

**Theorem 9 (Perov's fixed point theorem)**

Let (*X*, *d*) with *d*(*x*, *y*) ∈ ℝ^*m*^ be a complete generalized metric space and *A* : *X* → *X* an operator. One supposes that there exists a matrix *Q* ∈ *M*_*mm*_(ℝ_+_), such that

- *d*(*A*(*x*), *A*(*y*)) ≤ *Qd*(*x*, *y*), for all *x*, *y* ∈ *X*;
- *Q* ^*n*^ → 0 as *n* → *∞*.

Then

- *F*_*A*_ = {x*};
- *A* ^*n*^(*x*) = x*  as  *n* → *∞*, ∀*x* ∈ *X*;
- *d*(*A* ^*n*^(*x*), x*)≤(*I* − *Q*)^−1^ *Q* ^*n*^ *d*(*x*_0_, *A*(*x*_0_)).

## 3. Main Results

In this section, we present existence, uniqueness, and data dependence (monotony, continuity, and differentiability with respect to parameter) results of solution for the Cauchy problem (6)-(7).

### 3.1. Existence and Uniqueness

Using Perov's fixed point theorem, we obtain existence and uniqueness theorem for the solution of the problem (6)-(7).

**Theorem 10**

One supposes that

- the conditions (C_1_)–(C_3_) are satisfied;
- *Q* ^*n*^ → 0 as *n* → *∞*, where $Q=\left(b-a\right)\left(\begin{array}{cc} 0 & 1\\ L_{1} & L_{2} \end{array}\right)$ .

Then,

(a) the problem (6)-(7) has a unique solution $\left(\begin{array}{c} x^{*}\\ z^{*} \end{array}\right)\in C^{1}([a,b],{\mathbb{R}}^{2})$ ;
(b) for all $\left(\begin{array}{c} x^{0}\\ z^{0} \end{array}\right)\in C^{1}([a,b],{\mathbb{R}}^{2})$ , the sequence ${\left(\begin{array}{c} x^{n}\\ z^{n} \end{array}\right)}_{n\in \mathbb{N}}$ defined by $\left(\begin{array}{c} x^{n+1}\\ z^{n+1} \end{array}\right)=B\left(\begin{array}{c} x^{n}\\ z^{n} \end{array}\right)$ converges uniformly to $\left(\begin{array}{c} x^{*}\\ z^{*} \end{array}\right)$ , for all *t* ∈ [*a*, *b*], and
(17) ||(xnzn)−(x∗z∗)|| ≤(I−Q)−1Qn||(x0z0)−(x1z1)||;
(c) the operator *B* is Picard operator in $\left(C([a-h,b],{\mathbb{R}}^{2}),\overset{unif}{\to }\right)$ ;
(d) the operator *E* is weakly Picard operator in $\left(C([a-h,b],{\mathbb{R}}^{2}),\overset{unif}{\to }\right)$ .

**Proof**

Consider on the space *X*≔*C*([*a* − *h*, *b*], ℝ^2^) the norm

(18) $$\begin{array}{c} ||\left(\begin{array}{c} u_{1}\\ v_{1} \end{array}\right)-\left(\begin{array}{c} u_{2}\\ v_{2} \end{array}\right)||≔\max\left(\begin{array}{c} \left|u_{1}-u_{2}\right|\\ \left|v_{1}-v_{2}\right| \end{array}\right), \end{array}$$

which endows *X* with the uniform convergence.

Let $X_{\left(\begin{array}{c} \chi \\ \chi^{\prime } \end{array}\right)}=\{(\begin{array}{c} x\\ z \end{array})\in X|{\left(\begin{array}{c} x\\ z \end{array}\right)|}_{\left[a-h,a\right]}=(\begin{array}{c} \chi \\ \chi^{\prime } \end{array})$ , for $\left(\begin{array}{c} \chi \\ \chi^{\prime } \end{array})\in C([a-h,a],{\mathbb{R}}^{2})\right\}$ . Then $X=\cup_{\left(\begin{array}{c} \chi \\ \chi^{\prime } \end{array})\in C([a-h,a],{\mathbb{R}}^{2}\right)}X_{\left(\begin{array}{c} \chi \\ \chi^{\prime } \end{array}\right)}$ is a partition of *X*, and from [12] we have

1.
$B(X)\subset X_{\left(\begin{array}{c} \chi \\ \chi^{\prime } \end{array}\right)},\;B(X_{\left(\begin{array}{c} \chi \\ \chi^{\prime } \end{array}\right)})\subset X_{\left(\begin{array}{c} \chi \\ \chi^{\prime } \end{array}\right)}$ ;
2.
$B{|}_{X_{\left(\begin{array}{c} \chi \\ \chi^{\prime } \end{array}\right)}}=E{|}_{X_{\left(\begin{array}{c} \chi \\ \chi^{\prime } \end{array}\right)}}$ .

On the other hand, for *t* ∈ [*a* − *h*, *a*]∪[*a*, *b*]

(19) ||(B1(x1z1)B2(x1z1))−(B1(x2z2)B2(x2z2))|| ≤(b−a)(01L1L2)||(x1z1)−(x2z2)||,

whence *B* is a contraction in (*X*, ||·||) with $Q=\left(b-a\right)\left(\begin{array}{cc} 0 & 1\\ L_{1} & L_{2} \end{array}\right)$ . Applying Perov's theorem we obtain (a), (b), and (c). Moreover, the operator *B* is *c*-PO and *E* is *c*-WPO with $c={\left[1-(b-a)(\begin{array}{cc} 0 & 1\\ L_{1} & L_{2} \end{array})\right]}^{-1}$ .

### 3.2. Inequalities of Čaplygin Type

Now we establish the Čaplygin type inequalities.

**Theorem 11**

One supposes that

(i) the conditions (a), (b), and (c) in Theorem 10 are satisfied;
(ii)
$u_{i},v_{i},{\tilde{u}}_{i},{\tilde{v}}_{i}\in \mathbb{R}$ , $\left(\begin{array}{c} u_{i}\\ v_{i} \end{array}\right)\leq \left(\begin{array}{c} {\tilde{u}}_{i}\\ {\tilde{v}}_{i} \end{array}\right)$ ,  *i* = 1,2 imply that
(20) f(t,u1,v1,u2,v2)≤f(t,u~1,v~1,u~2,v~2),∀t∈[a,b].

Let $\left(\begin{array}{c} x^{*}\\ z^{*} \end{array}\right)$ be a solution of (6) and $\left(\begin{array}{c} y^{*}\\ w^{*} \end{array}\right)$ a solution of the system

(21) y′(t)≤w(t), t∈[a,b],w′(t)≤f(t,y(t),w(t),y(t−h),w(t−h)),t∈[a,b].

Then

(22) $$\begin{array}{c} {\left(\begin{array}{c} y^{*}\\ w^{*} \end{array}\right)|}_{\left[a-h,a\right]}\leq {\left(\begin{array}{c} x^{*}\\ z^{*} \end{array}\right)|}_{\left[a-h,a\right]}\;\;implies\;that\;\left(\begin{array}{c} y^{*}\\ w^{*} \end{array}\right)\leq \left(\begin{array}{c} x^{*}\\ z^{*} \end{array}\right). \end{array}$$

**Proof**

We have that

(23) $$\begin{array}{c} \left(\begin{array}{c} x^{*}\\ z^{*} \end{array}\right)=E\left(\begin{array}{c} x^{*}\\ z^{*} \end{array}\right),\;\;\left(\begin{array}{c} y^{*}\\ w^{*} \end{array}\right)\leq E\left(\begin{array}{c} y^{*}\\ w^{*} \end{array}\right). \end{array}$$

From Theorem 10, (c), *E* is a weakly Picard operator. From condition (ii), we obtain that *E* ^*∞*^ is increasing [11]. So

(24) (y∗w∗)≤E∞(y∗w∗)=E∞(y~∗w~∗)≤E∞(x~∗z~∗)=(x∗z∗),

where $(\begin{array}{c} {\tilde{x}}^{*}\\ {\tilde{z}}^{*} \end{array})\in X_{{\left(\begin{array}{c} x\\ z \end{array}\right)|}_{\left[a-h,a\right]}}$ .

### 3.3. Data Dependence: Monotony

In this subsection, we study the monotony of the solution of the problem (6)-(7) with respect to *φ* and *f*.

**Theorem 12 (comparison theorem)**

Let *f*_*i*_ ∈ *C*([*a*, *b*] × ℝ^4^, ℝ),  *i* = 1,2, 3, be as in Theorem 10. One supposes that

- *f*_1_ ≤ *f*_2_ ≤ *f*_3_;
- *f*_2_(*t*, ·, ·, ·, ·) : ℝ^4^ → ℝ is increasing, ∀*t* ∈ [*a*, *b*].

Let $\left(\begin{array}{c} x_{i}^{*}\\ z_{i}^{*} \end{array}\right)$ be a solution of the system

(25) x′(t)=z(t), t∈[a,b],z′(t)=fi(t,x(t),z(t),x(t−h),z(t−h)),t∈[a,b].

Then

(26) $$\begin{array}{c} {\left(\begin{array}{c} x_{1}^{*}\\ z_{1}^{*} \end{array}\right)|}_{\left[a-h,a\right]}\leq {\left(\begin{array}{c} x_{2}^{*}\\ z_{2}^{*} \end{array}\right)|}_{\left[a-h,a\right]}\leq {\left(\begin{array}{c} x_{3}^{*}\\ z_{3}^{*} \end{array}\right)|}_{\left[a-h,a\right]} \end{array}$$

imply that $\begin{array}{c} {\left(\begin{array}{c} x_{1}^{*}\\ z_{1}^{*} \end{array}\right)|}_{\left[a-h,a\right]}\leq {\left(\begin{array}{c} x_{2}^{*}\\ z_{2}^{*} \end{array}\right)|}_{\left[a-h,a\right]}\leq {\left(\begin{array}{c} x_{3}^{*}\\ z_{3}^{*} \end{array}\right)|}_{\left[a-h,a\right]}. \end{array}$

**Proof**

We consider the operators *E*_*i*_ corresponding to each system (25). The operators *E*_*i*_, *i* = 1,2, 3 are weakly Picard operators. Taking into consideration the condition (ii), *E*_2_ is increasing. From (i) we have *E*_1_ ≤ *E*_2_ ≤ *E*_3_. On the other hand, we have that

(27) $$\begin{array}{c} \left(\begin{array}{c} x_{i}^{*}\\ z_{i}^{*} \end{array}\right)=E_{i}^{\infty }\left(\begin{array}{c} {\tilde{x}}_{i}^{*}\\ {\tilde{z}}_{i}^{*} \end{array}\right),\;i=1,2,3, \end{array}$$

where $\left(\begin{array}{c} {\tilde{x}}^{*}\\ {\tilde{z}}^{*} \end{array}\right)\in X_{{\left(\begin{array}{c} x\\ z \end{array}\right)|}_{\left[a-h,a\right]}}$ . The proof follows from the abstract comparison Lemma (see [11]).

### 3.4. Data Dependence: Continuity

Consider the problem (6)-(7) with the dates *f*_*i*_∈*C*([*a*, *b*] × ℝ^4^, ℝ),  *i* = 1,2 and suppose that *f*_*i*_ satisfy the conditions from Theorem 10 with the same Lipshitz constants. We obtain the data dependence result.

**Theorem 13**

Let *f*_*i*_ ∈ *C*([*a*, *b*] × ℝ^4^, ℝ), *φ*_*i*_ ∈ *C*([*a* − *h*, *a*], ℝ),  *i* = 1,2, be as in Theorem 10. One supposes that

(i) there exists *η*_1_, *η*_2_ > 0 such that
(28) $$\begin{array}{c} \left|\left(\begin{array}{c} \varphi_{1}\\ \varphi_{1}^{\prime } \end{array}\right)\left(t\right)-\left(\begin{array}{c} \varphi_{2}\\ \varphi_{2}^{\prime } \end{array}\right)\left(t\right)\right|\leq \left(\begin{array}{c} \eta_{1}\\ \eta_{2} \end{array}\right),\;\forall t\in \left[a-h,a\right]; \end{array}$$
(ii) there exists *η*_3_ > 0 such that
(29) |f1(t,u1,u2,u3,u4)−f2(t,u1,u2,u3,u4)|≤η3,∀t∈[a,b],  ui∈ℝ,i=1,4¯.

Then

(30) ||(x∗z∗)(·,φ1,φ1′,f1)−(x∗z∗)(·,φ2,φ2′,f2)|| ≤(I−Q)−1(η1η2+(b−a)η3),

where $\left(\begin{array}{c} x^{*}\\ z^{*} \end{array}\right)(\cdot ,\varphi ,\varphi^{\prime },f)$ denote the unique solution of (6)-(7).

**Proof**

Consider the operators *B*_*φ*_*i*_,*f*_*i*__, *i* = 1,2. From Theorem 10, it follows that

(31) ||Bφ1,f1(x1z1)−Bφ1,f1(x2z2)|| ≤Q||(x1z1)−(x2z2)||, ∀(xizi)∈X.

Additionally,

(32) $$\begin{array}{c} ||B_{\varphi_{1},f_{1}}\left(\begin{array}{c} x\\ z \end{array}\right)-B_{\varphi_{2},f_{2}}\left(\begin{array}{c} x\\ z \end{array}\right)||\leq \left(\begin{array}{c} \eta_{1}\\ \eta_{2}+\left(b-a\right)\eta_{3} \end{array}\right). \end{array}$$

Thus,

(33) ||(x∗z∗)(·,φ1,φ1′,f1)  −(x∗z∗)(·,φ2,φ2′,f2)|| =||Bφ1,f1((x∗z∗)(·,φ1,φ1′,f1))   −Bφ2,f2((x∗z∗)(·,φ2,φ2′,f2))|| ≤||Bφ1,f1((x∗z∗)(·,φ1,φ1′,f1))   −Bφ1,f1((x∗z∗)(·,φ2,φ2′,f2))||  +||Bφ1,f1((x∗z∗)(·,φ2,φ2′,f2))    −Bφ2,f2((x∗z∗)(·,φ2,φ2′,f2))|| ≤Q||(x∗z∗)(·,φ1,φ1′,f1)    −(x∗z∗)(·,φ2,φ2′,f2)||+(η1η2+(b−a)η3),

and since *Q* ^*n*^ → *∞*, as *n* → *∞* implies that (*I* − *Q*)^−1^ ∈ *M*_22_(ℝ_+_), we finally obtain

(34) ||(x∗z∗)(·,φ1,φ1′,f1)−(x∗z∗)(·,φ2,φ2′,f2)|| ≤(I−Q)−1(η1η2+(b−a)η3).

### 3.5. Data Dependence: Differentiability

Consider the following differential system with parameter:

(35) x′(t)=z(t), t∈[a,b],z′(t)=f(t,x(t),z(t),x(t−h),z(t−h);λ),t∈[a,b], λ∈J,

with the initial conditions

(36) $$\begin{array}{c} x\left(t\right)=\varphi \left(t\right),\;t\in \left[a-h,a\right],\\ z\left(t\right)=\varphi^{\prime }\left(t\right),\;t\in \left[a-h,a\right], \end{array}$$

where *J* ⊂ ℝ is a compact interval.

Suppose that the following conditions are satisfied:

(C_1_) *a* < *b*, *h* > 0, *J* ⊂ ℝ a compact interval;
(C_2_) *f* ∈ *C*([*a*, *b*] × ℝ^4^ × *J*, ℝ);
(C_3_) *φ* ∈ *C* ^1^([*a* − *h*, *a*], ℝ);
(C_4_) there exists *L*_*f*_ > 0, such that
(37) |∂f(t,u1,u2,u3,u4;λ)∂ui|≤L1,u1,u3∈ℝ,i=1,3, λ∈J;|∂f(t,u1,u2,u3,u4;λ)∂ui|≤L2,u2,u4∈ℝ,i=2,4, λ∈J;
(C_5_) for $Q=\left(b-a\right)\left(\begin{array}{cc} 0 & 1\\ L_{1} & L_{2} \end{array}\right)$ , we have *Q* ^*n*^ → 0 as *n* → *∞*.

Then, from Theorem 10, we have that the problem (6)-(7) has a unique solution, $\left(\begin{array}{c} x^{*}\\ z^{*} \end{array}\right)\in C^{1}([a,b],{\mathbb{R}}^{2})$ . We prove that $\left(\begin{array}{c} x^{*}\\ z^{*} \end{array}\right)\in C^{1}(J,{\mathbb{R}}^{2})$ , ∀*t* ∈ [*a* − *h*, *b*].

For this we consider the system

(38) x′(t;λ)=z(t;λ), t∈[a,b], λ∈J,z′(t;λ)=f(t,x(t;λ),z(t;λ),   x(t−h;λ),z(t−h;λ);λ),         t∈[a,b], λ∈J,

with $\left(\begin{array}{c} x^{*}\\ z^{*} \end{array}\right)\in C([a-h,b]\times J,{\mathbb{R}}^{2})\cap C^{1}([a,b]\times J,{\mathbb{R}}^{2})$ .

**Theorem 14**

Consider the problem (38)–(36) and suppose the conditions (C_1_)–(C_5_) hold. Then,

- Equations (38)–(36) have a unique solution $\left(\begin{array}{c} x^{*}\\ z^{*} \end{array}\right)(\cdot ,\lambda )$ , in *C*([*a* − *h*, *b*] × *J*, ℝ^2^);
-
$\left(\begin{array}{c} x^{*}\\ z^{*} \end{array}\right)(\cdot ,\lambda )\in C^{1}(J,{\mathbb{R}}^{2})$ , ∀*t* ∈ [*a* − *h*, *b*].

**Proof**

The problem (38)–(36) is equivalent with the following functional-integral system:

(39) (xz)(t;λ)=(φ(a)+∫atz(s;λ)dsφ′(a)+∫atf(s,x(s;λ),z(s;λ),x(s−h;λ),z(s−h;λ);λ)ds),

for *t* ∈ [*a*, *b*] and

(40) $$\begin{array}{c} \left(\begin{array}{c} x\\ z \end{array}\right)\left(t;\lambda \right)=\left(\begin{array}{c} \varphi \\ \varphi^{\prime } \end{array}\right)\left(t\right),\;\text{for}\;t\in \left[a-h,a\right]. \end{array}$$

Now let us take the operator *A* : *C*([*a* − *h*, *b*] × *J*, ℝ^2^) → *C*([*a* − *h*, *b*] × *J*, ℝ^2^), defined by

(41) A(xz)(t;λ)≔(A1(xz)A2(xz))≔the right hand side of (39),for  t∈[a,b],A(xz)(t;λ)≔(A1(xz)A2(xz))≔the right hand side of (40),for  t∈[a−h,a].

Let *X* = *C*([*a* − *h*, *b*] × *J*, ℝ^2^).

It is clear, from the proof of the Theorem 10, that in the condition (C_1_)–(C_5_), the operator *A* : (*X*, ||·||)→(*X*, ||·||) is Picard operator.

Let $\left(\begin{array}{c} x^{*}\\ z^{*} \end{array}\right)$ be the unique fixed point of *A*.

Supposing that there exists $\left(\begin{array}{c} \partial x^{*}/\partial \lambda \\ \partial z^{*}/\partial \lambda \end{array}\right)$ , from (39)-(40), we obtain that

(42) ∂x∗∂λ=∫at∂z(s;λ)∂λds,∂z∗∂λ=∫at((∂f(s,x(s;λ),z(s;λ),x(s−h;λ),   z(s−h;λ);λ))×(∂u1)−1)∂x(s;λ)∂λds +∫at((∂f(s,x(s;λ),z(s;λ),x(s−h;λ),    z(s−h;λ);λ))×(∂u2)−1)∂z(s;λ)∂λds +∫at((∂f(s,x(s;λ),z(s;λ),x(s−h;λ),    z(s−h;λ);λ))×(∂u3)−1)∂x(s−h;λ)∂λds +∫at((∂f(s,x(s;λ),z(s;λ),x(s−h;λ),    z(s−h;λ);λ))×(∂u4)−1)∂z(s−h;λ)∂λds +∫at((∂f(s,x(s;λ),z(s;λ),x(s−h;λ),    z(s−h;λ);λ))×(∂λ)−1)ds,

for all *t* ∈ [*a*, *b*], *λ* ∈ *J*.

This relation suggests that we consider the following operator:

(43) $$\begin{array}{c} C:X\times X⟶X,\\ \left(\left(\begin{array}{c} x_{12}\\ z_{12} \end{array}\right),\left(\begin{array}{c} u_{13}\\ u_{24} \end{array}\right)\right)⟶C\left(\left(\begin{array}{c} x_{12}\\ z_{12} \end{array}\right),\left(\begin{array}{c} u_{13}\\ u_{24} \end{array}\right)\right), \end{array}$$

where $C\left(\left(\begin{array}{c} x_{12}\\ z_{12} \end{array}),(\begin{array}{c} u_{13}\\ u_{24} \end{array}\right)\right)(t;\lambda )=0$ for *t* ∈ [*a* − *h*, *a*], *λ* ∈ *J*, and

(44) C((x12z12),(u13u24))(t;λ)≔(C1((x12z12),(u13u24))C2((x12z12),(u13u24))),

where

(45) C1((x12z12),(u13u24))(t;λ)≔∫at∂z(s;λ)∂λds,C2((x12z12),(u13u24))(t;λ) ≔∫at((∂f(s,x(s;λ),z(s;λ),x(s−h;λ),    z(s−h;λ);λ))×(∂u1)−1)u1(s;λ)ds  +∫at((∂f(s,x(s;λ),z(s;λ),x(s−h;λ),     z(s−h;λ);λ))×(∂u2)−1)u2(s;λ)ds  +∫at((∂f(s,x(s;λ),z(s;λ),x(s−h;λ),     z(s−h;λ);λ))×(∂u3)−1)u3(s−h;λ)ds  +∫at((∂f(s,x(s;λ),z(s;λ),x(s−h;λ),     z(s−h;λ);λ))×(∂u4)−1)u4(s−h;λ)ds  +∫at((∂f(s,x(s;λ),z(s;λ),x(s−h;λ),     z(s−h;λ);λ))×(∂λ)−1)ds,

for *t* ∈ [*a*, *b*],  *λ* ∈ *J*. Here we use the notations *u*_1_(*s*; *λ*)≔∂*x*(*s*; *λ*)/∂*λ*, *u*_2_(*s*; *λ*)≔∂*z*(*s*; *λ*)/∂*λ*, *u*_3_(*s* − *h*; *λ*)≔∂*x*(*s* − *h*; *λ*)/∂*λ*, and *u*_4_(*s* − *h*; *λ*)≔∂*z*(*s* − *h*; *λ*)/∂*λ*.

In this way, we have the triangular operator *D* : *X* × *X* → *X* × *X*, $\left(\left(\begin{array}{c} x_{12}\\ z_{12} \end{array}\right),\left(\begin{array}{c} u_{13}\\ u_{24} \end{array}\right)\right)\to \left(A\left(\begin{array}{c} x_{12}\\ z_{12} \end{array}\right),C\left(\left(\begin{array}{c} x_{12}\\ z_{12} \end{array}\right),\left(\begin{array}{c} u_{13}\\ u_{24} \end{array}\right)\right)\right)$ , where *A* is Picard operator and $C\left(\left(\begin{array}{c} x_{12}\\ z_{12} \end{array}\right),\left(\begin{array}{c} \cdot \\ \cdot \end{array}\right)\right):X\to X$ is *Q*_*C*_-contraction with $Q_{C}=(b-a)\left(\begin{array}{cc} 0 & 1\\ L_{1} & L_{2} \end{array}\right)$ .

From Theorem 8 the operator *D* is Picard operator; that is, the sequences

(46) $$\begin{array}{c} \left(\begin{array}{c} x_{12}^{n+1}\\ z_{12}^{n+1} \end{array}\right)≔A\left(\begin{array}{c} x_{12}^{n}\\ z_{12}^{n} \end{array}\right),\\ \left(\begin{array}{c} u_{13}^{n+1}\\ u_{24}^{n+1} \end{array}\right)≔C\left(\left(\begin{array}{c} x_{12}^{n}\\ z_{12}^{n} \end{array}\right),\left(\begin{array}{c} u_{13}^{n}\\ u_{24}^{n} \end{array}\right)\right), \end{array}$$

*n* ∈ *ℕ*, converge uniformly, with respect to *t* ∈ [*a* − *h*, *b*], *λ* ∈ *J*, to $\left(\left(\begin{array}{c} x_{12}^{n}\\ z_{12}^{n} \end{array}\right),\left(\begin{array}{c} u_{13}^{n}\\ u_{24}^{n} \end{array}\right)\right)\in F_{D}$ , for all $\left(\begin{array}{c} x_{12}^{0}\\ z_{12}^{0} \end{array}\right),\left(\begin{array}{c} u_{13}^{0}\\ u_{24}^{0} \end{array}\right)\in X$ .

If we take

(47) $$\begin{array}{c} \left(\begin{array}{c} x_{12}^{0}\\ z_{12}^{0} \end{array}\right)=\left(\begin{array}{c} 0\\ 0 \end{array}\right),\\ \left(\begin{array}{c} u_{13}^{0}\\ u_{24}^{0} \end{array}\right)=\left(\begin{array}{c} \frac{\partial x_{12}^{0}}{\partial \lambda }\\ \frac{\partial z_{12}^{0}}{\partial \lambda } \end{array}\right)=\left(\begin{array}{c} 0\\ 0 \end{array}\right), \end{array}$$

then

(48) $$\begin{array}{c} \left(\begin{array}{c} u_{13}^{1}\\ u_{24}^{1} \end{array}\right)=\left(\begin{array}{c} \frac{\partial x_{12}^{1}}{\partial \lambda }\\ \frac{\partial z_{12}^{1}}{\partial \lambda } \end{array}\right). \end{array}$$

By induction we prove that

(49) $$\begin{array}{c} \left(\begin{array}{c} u_{13}^{n}\\ u_{24}^{n} \end{array}\right)=\left(\begin{array}{c} \frac{\partial x_{12}^{n}}{\partial \lambda }\\ \frac{\partial z_{12}^{n}}{\partial \lambda } \end{array}\right),\;\forall n\in \mathbb{N}. \end{array}$$

So, $\left(\begin{array}{c} x_{12}^{n}\\ z_{12}^{n} \end{array}\right)\overset{\text{unif}}{⟶}\left(\begin{array}{c} x_{12}^{*}\\ z_{12}^{*} \end{array}\right)$ , as *n* → *∞*, and $\left(\begin{array}{c} \partial x_{12}^{n}/\partial \lambda \\ \partial z_{12}^{n}/\partial \lambda \end{array}\right)\overset{\text{unif}}{⟶}\left(\begin{array}{c} u_{13}^{*}\\ u_{24}^{*} \end{array}\right)$ , as *n* → *∞*.

From a Weierstrass argument we get that there exists

(50) $$\begin{array}{c} \left(\begin{array}{c} \frac{\partial x_{12}^{*}}{\partial \lambda }\\ \frac{\partial z_{12}^{*}}{\partial \lambda } \end{array}\right),\;i=1,2,\\ \left(\begin{array}{c} \frac{\partial x_{12}^{*}}{\partial \lambda }\\ \frac{\partial z_{12}^{*}}{\partial \lambda } \end{array}\right)=\left(\begin{array}{c} u_{13}^{*}\\ u_{24}^{*} \end{array}\right). \end{array}$$

### 3.6. Ulam-Hyers Stability

We start this section by presenting the Ulam-Hyers stability concept (see [15, 16]). For *f* ∈ *C*([*a*, *b*] × ℝ^4^, ℝ), *ɛ* > 0, and *ψ* ∈ *C*([*a* − *h*, *b*], ℝ_+_), *h* > 0, we consider the system

(51) x′(t)=z(t), t∈[a,b],z′(t)=f(t,x(t),z(t),x(t−h),z(t−h)),t∈[a,b]

and the following inequations

(52) |x′(t)−z(t)|≤ɛ, t∈[a,b],|z′(t)−f(t,x(t),z(t),x(t−h),z(t−h))|≤ɛ,t∈[a,b],

(53) |x′(t)−z(t)|≤ψ(t), t∈[a,b],|z′(t)−f(t,x(t),z(t),x(t−h),z(t−h))|≤ψ(t),t∈[a,b].

**Definition 15**

The system (51) is Ulam-Hyers stable if there exists a real number *c* > 0 such that for each *ɛ* > 0 and for each solution $\left(\begin{array}{c} y\\ w \end{array}\right)\in C^{2}([a-h,b],{\mathbb{R}}^{2})$ of (52) there exists a solution $\left(\begin{array}{c} x\\ z \end{array}\right)\in C^{2}([a-h,b],{\mathbb{R}}^{2})$ of (51) with

(54) $$\begin{array}{c} \left|\left(\begin{array}{c} y\\ w \end{array}\right)\left(t\right)-\left(\begin{array}{c} x\\ z \end{array}\right)\left(t\right)\right|\leq cɛ,\;\forall t\in \left[a-h,b\right]. \end{array}$$

**Theorem 16**

One supposes that

- the conditions (C_1_)–(C_3_) are satisfied;
- *Q* ^*n*^ → 0 as *n* → *∞*, where $Q=(b-a)\left(\begin{array}{cc} 0 & 1\\ L_{1} & L_{2} \end{array}\right)$ .

Then the system (6) is Ulam-Hyers stable.

**Proof**

The system (6) is equivalent with the functional integral system (10). We consider the operator *E* : *C*([*a* − *h*, *b*], ℝ^2^) → *C*([*a* − *h*, *b*], ℝ^2^), defined by $E\left(\begin{array}{c} x\\ z \end{array}\right)(t)≔$ the right hand side of (10), for *t* ∈ [*a* − *h*, *b*]. So

(55) $$\begin{array}{c} \left(\begin{array}{c} x\\ z \end{array}\right)=E\left(\begin{array}{c} x\\ z \end{array}\right)\left(t\right),\;t\in \left[a-h,b\right]. \end{array}$$

From Theorem 10, *E* is *c*-WPO with $c={\left[1-(b-a)(\begin{array}{cc} 0 & 1\\ L_{1} & L_{2} \end{array})\right]}^{-1}$ . Applying Theorem 7 we obtain that (6) is Ulam-Hyers stable.

**Remark 17**

Another proof for the above theorem can be done using Gronwall lemma [8, 14–17].

## References

[1] Kolmanovskiĭ V, Myshkis A (1992). *Applied Theory of Functional-Differential Equations*.

[2] Pinney E (1958). *Ordinary Difference-Differential Equations*.

[3] Guljaev VV, Dmitriev AS, Kislov VE (1985). Strange attractors in the circle: selfoscillating systems. *Doklady Akademii Nauk SSSR*.

[4] Kolmanovskiĭ VB, Nosov VR (1986). *Stability of Functional-Differential Equations*.

[5] Ilea VA, Otrocol D (2009). On a D. V. Ionescu's problem for functional-differential equations. *Fixed Point Theory*.

[6] Olaru IM (2010). An integral equation via weakly Picard operators. *Fixed Point Theory*.

[7] Olaru IM (2010). Data dependence for some integral equations. *Studia. Universitatis Babeş-Bolyai. Mathematica*.

[8] Otrocol D, Ilea V (2013). Ulam stability for a delay differential equation. *Central European Journal of Mathematics*.

[9] Precup R (2009). The role of matrices that are convergent to zero in the study of semilinear operator systems. *Mathematical and Computer Modelling*.

[10] Rus IA (1979). *Principles ans Applications of the Fixed Point Theory*.

[11] Rus IA (2001). *Generalized contractions and applications*.

[12] Rus IA (2002). Functional-differential equations of mixed type, via weakly Picard operators. *Seminar on Fixed Point Theory Cluj-Napoca*.

[13] Rus IA (2003). Picard operators and applications. *Scientiae Mathematicae Japonicae*.

[14] Rus IA (2009). Gronwall lemmas: ten open problems. *Scientiae Mathematicae Japonicae*.

[15] Rus IA (2009). Ulam stability of ordinary differential equations. *Studia. Universitatis Babeş-Bolyai. Mathematica*.

[16] Rus IA (2009). Remarks on Ulam stability of the operatorial equations. *Fixed Point Theory*.

[17] Rus IA, Rassias TM, Brzdek J (2011). Ulam stability of the operatorial equations. *Functional Equations in Mathematical Analysis*.

[18] Perov AI, Kibenko AV (1966). On a general method to study boundary value problems. *Izvestiya Akademii Nauk SSSR. Seriya Matematicheskaya*.

